# Hypermethylation of HIC2 is a potential prognostic biomarker and tumor suppressor of glioma based on bioinformatics analysis and experiments

**DOI:** 10.1111/cns.14093

**Published:** 2023-01-17

**Authors:** Feifei Luo, Yifu Liao, Endong Cao, Yong Yang, Kai Tang, Dexiang Zhou, Dong Zhou, Haiping Cai

**Affiliations:** ^1^ Cancer Epigenetics Laboratory, Department of Clinical Oncology, State Key Laboratory of Oncology in South China Sir YK Pao Center for Cancer and Li Ka Shing Institute of Health Sciences, The Chinese University of Hong Kong Hong Kong China; ^2^ Department of Neurology Guangdong Provincial People's Hospital (Guangdong Academy of Medical Sciences), Southern Medical University Guangzhou China; ^3^ Department of Neurosurgery Guangdong Provincial People's Hospital (Guangdong Academy of Medical Sciences), Southern Medical University Guangzhou China

**Keywords:** DNA methylation, glioma, HIC2, prognostic biomarkers, tumor suppressor gene

## Abstract

**Introduction:**

Glioma is the most common primary tumor in the central nervous system, and prognostic biomarkers are still lacking. HIC ZBTB transcriptional repressor 2 (HIC2) is a hypermethylated gene that plays an important functional role in cardiac development. However, the actual role of HIC2 in glioma progression remains unclear. This study aimed to investigate the function of HIC2 and whether it could be a prognostic biomarker in glioma.

**Methods:**

The DNA methylation and mRNA expression profiles of HIC2 were downloaded from public databases. The prognostic prediction ability and mechanism research of HIC2 were evaluated.

**Results:**

We found that HIC2 was hypermethylated and expressed at low levels in glioma samples. Hypermethylation and low expression of HIC2 predicted poor prognosis. Multivariate Cox regression analysis suggested that HIC2 was an independent prognostic factor for gliomas. Co‐IP assays demonstrated that HIC2 interacts with RNF44, and dual‐luciferase reporter assays and ChIP assays revealed that HIC2 transcriptionally inhibits PTPRN2 expression.

**Conclusions:**

Our findings suggest that HIC2 represents a tumor suppressor gene and prognostic biomarker for glioma progression and that overexpression of HIC2 inhibits the proliferation of glioma in vitro and in vivo by interacting with RNF44 and PTPRN2.

## Graphical abstract

The DNA methylation and mRNA expression profiles of HIC2 were downloaded from the Chinese Glioma Genome Atlas (CGGA), The cancer genome atlas (TCGA), The Genotype‐Tissue Expression (GTEx) and gene expression omnibus (GEO) databases. We found that HIC2 was hypermethylated and expressed at low levels in glioma samples. Hypermethylation and low expression of HIC2 predicted poor prognosis. Multivariate Cox regression analysis suggested that HIC2 was an independent prognostic factor for gliomas. Furthermore, GO, KEGG, and GSEA analyses revealed that HIC2 expression was associated with cell growth, immune responses, and the mTOR signaling pathway. HIC2 expression was correlated with immune cell infiltration. Co‐IP assays demonstrated that HIC2 interacts with RNF44, and dual‐luciferase reporter assays and ChIP assays revealed that HIC2 transcriptionally inhibits PTPRN2 expression.

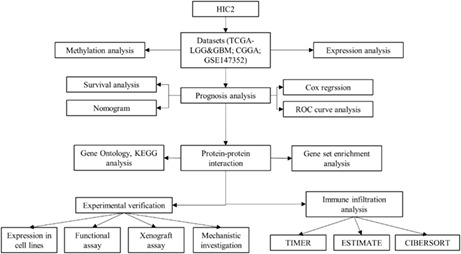

## 1 INTRODUCTION

Glioma is the most common primary malignant brain tumor in adults, representing approximately 81% of malignant brain tumors.^1^, ^2^ Glioblastoma has the poorest overall survival, with only 0.05% to 4.7% of patients surviving 5 years past their diagnosis.^3^, ^4^ Currently, conventional treatment for gliomas includes surgical resection followed by a combination of radiotherapy or chemotherapy comprised of temozolomide (TMZ). However, due to inherent or acquired resistance to traditional combination therapy, inevitable relapse or malignant progression eventually leads to an unfavorable prognosis in glioma patients, especially GBM patients.^5^, ^6^ Identification of new biomarkers of glioma may lead to new evidence regarding risk and prognosis.

HIC ZBTB transcriptional repressor 2 (HIC2) is also known as HRG22, ZBTB30, ZNF907 or hypermethylated in cancer 2. HIC2 located in the nucleoplasm and enables protein C terminal binding activity, which was predicted to be involved in the regulation of transcription by RNA polymerase II. Specifically, HIC2 belongs to the Krueppel C2H2‐type zinc‐finger protein family, HIC subfamily and POK family, meaning its protein isoforms contain the BTB/POZ domain for interacting with other proteins at its N‐terminus and the C2H2 zinc finger domain that recognize and bind specific DNA sequences (GGCA) at its C‐terminus.^7^, ^8^, ^9^ In 2001, Deltour S et al.^9^ first reported that HIC2 shares high sequence homology with HIC1 and is located on chromosome 22q11.2. Epigenetic silencing and downregulation of the HIC1 gene have been frequently observed in different types of human malignancies, including gastric and liver cancers, esophageal cancers, and breast cancers.^10^, ^11^ To date, some studies have reported that HIC2 plays a role in embryogenesis by suppressing fetal genes for normal development of the heart and circulatory system.^12^, ^13^ Despite this knowledge, the structural and biochemical characteristics and functions of HIC2 as a transcription factor remain unknown. In particular, the role of HIC2 in tumorigenesis remains unclear.

In this study, after a comprehensive bioinformatics and experimental analysis of the role of HIC2 in glioma, we found that HIC2 was epigenetically silenced by hypermethylation in its promoter and low expression of HIC2 indicated an unfavorable prognosis.

## 2 METHODS

### 2.1 Data acquisition and processing

RNA‐sequence data, microarray data, and their corresponding clinical information were downloaded from three databases: TCGA and GTEx (http://xena.ucsc.edu/), CGGA (www.cgga.org.cn) and GEO (https://www.ncbi.nlm.nih.gov/gds), including 1819 samples from TCGA and GTEx (662 normal samples and 1157 glioma samples), 1218 samples from CGGA (20 normal samples and 1018 glioma samples), 118 samples from GEO GSE147352 (15 normal samples and 103 glioma samples), and 85 glioma samples from GSE4412. Clinical information in the datasets included age, sex, overall survival, WHO grade, IDH1 status, MGMT promoter methylation status, and 1p/19q status. Some samples with unavailable or unclear clinical information were removed. In addition, DNA methylation data for TCGA samples were downloaded.

### 2.2 Survival data analysis

Glioma samples were divided into high HIC2 expression and low HIC2 expression groups based on the median HIC2 expression level. Kaplan–Meier survival analysis was used to evaluate the difference in overall survival time between the two groups using the log‐rank test.

### 2.3 Cox regression analysis and risk score heatmap construction

The prognostic prediction ability of HIC2 expression, age, grade, IDH1 mutation, and 1p/19q status were examined using univariate and multivariate Cox regression based on samples from the TCGA and CGGA databases. Hazard ratios (HRs) and 95% confidence intervals (CIs) were calculated accordingly.

### 2.4 Construction of the prognostic nomogram and ROC curve

A nomogram based on HIC2 expression, survival status, and clinical characteristics was constructed using the R software package rms and was used to predict glioma patient prognosis. The deviation between the predicted probability and the actual outcome was visualized by constructing the calibration curves. A concordance index (C index) was used to measure the accuracy of nomogram prediction. The time‐dependent receiver operating characteristic (ROC) curve and area under the curve (AUC) were constructed using the R software package pROC.

### 2.5 Immune cell infiltration analysis

The correlation between HIC2 expression and immune cell infiltration in LGG and GBM was analyzed using the Tumor Immune Estimation Resource (TIMER 2.0) database. The R package EpiDISH was used to analyze the correlation between HIC2 methylation sites cg13558199, cg20944928, and cg22869804 and immune cell infiltration. The degree of immune cell infiltration was calculated using the ESTIMATE (https://bioinformatics.mdanderson.org/estimate) database based on the expression data.

### 2.6 Enrichment analysis

Potential HIC2‐associated functions were predicted with GO and KEGG analyses using the R software cluster profiler package (version 3.14.3). The TISIDB database (http://cis.hku.hk/TISIDB) was used to evaluate the correlation between HIC2 and immunomodulators. The protein–protein interaction network was predicted using the STRING database (www.string‐db.org/) and was visualized using Cytoscape software. The detection of the top 100 similar genes was performed by the GEPIA2 database (http://gepia2.cancer‐pku.cn/), and the intersection between the HIC2 top 100 similar genes and HIC2‐interacting genes was visualized using a Venn diagram in the R software Venn Diagram package.

### 2.7 In vitro and in vivo functional assays

Cell proliferation assays were used to evaluate the biological function of HIC2 in glioma cells. Briefly, 3000 cells were seeded into each well of 96‐well plates, and cell viability was assessed every 24 h for four consecutive days using a Cell Counting Kit‐8 (Dojindo, Kumamoto, Japan). Xenograft growth of glioma cells in mice was used to evaluate the role of HIC2 expression in glioma cell proliferation in vivo. Briefly, five‐week‐old female C57BL/6 mice (Nanjing, Jiangsu, China) were randomly divided into two groups (five per group). GL261 glioma cells with or without stable overexpression of HIC2 were implanted intracranial at a density of 1 × 10^5^ cells to establish orthotopic xenografts monitored by bioluminescence imaging (BLI) every 7 days. The survival time of mice‐bearing orthotopic xenografts was observed. This animal experiments were approved by the institutional ethics committee of Guangdong Provincial People's Hospital (Ethical approval number: KY‐X‐2022‐018‐02).

### 2.8 Western blot analysis

Western blot analysis was performed according to a standard protocol. Briefly, equal concentrations of protein (30 μg) were separated and transferred onto polyvinylidene difluoride membranes (EMD Millipore). The membranes were then probed with the appropriate primary antibody. All antibodies were as follows: anti‐HIC2 (ab167257, Abcam), anti‐HIC2 (PA5‐37293, Invitrogen), anti‐Flag (ab205606, Abcam), anti‐HA (ab236632, Abcam), anti‐Akt (#4691, CST), anti‐p‐Akt (#4060, CST), anti‐mTOR (#2983, CST), anti‐p‐mTOR (#5536, CST), anti‐p70S6K (#2708, CST), anti‐p‐p70S6K (#9234, CST), and anti‐GAPDH (60,004, Proteintech, IL, USA).

### 2.9 Lentivirus production, transduction, and plasmid construction

The H149 vector containing full‐length HIC2 was stably transfected into LN229 and U251 glioma cells using a Lenti‐Pac HIV Kit (GeneCopoeia, LT002) according to the manufacturer's instructions. pSLenti‐HIC2‐EGFP‐3xFLAG‐WPRE containing full‐length mouse HIC2 were purchased from Obio Technology (Shanghai) and stably transfected into GL261 mouse glioma cell. Stably transfected cells were further selected by culturing with media supplemented with 2 μg/ml puromycin (MCE, Monmouth Junction, NJ, USA). Expression of HIC2 in stably transfected glioma cells was detected by qRT–PCR using a Bio‐Rad CFX96 Real‐Time PCR System (Bio‐Rad Laboratories, Inc.) and western blot analysis. For plasmid construction, the Flag‐tagged RNF44 constructs and HA‐tagged HIC2 were cloned into the pcDNA3.1 vector. For intracranial glioma models imaging, GL261 glioma cells were transfected with lentivirus H113 to overexpress luciferase (pLenti CMV EGFP linker Luc PKG puro; Obio Technology, Shanghai).

### 2.10 Dual‐luciferase reporter assay and chromatin immunoprecipitation (ChIP) assay

The promoter region and the mutant (MT) forms (E1 and E2) of PTPRN2 were subcloned into a pGL4‐luc vector that contained the firefly luciferase gene (OBIO) to establish three constructs, PTPRN2 WT, Mut‐E1, and Mut‐E2. The pRL‐CMV vector containing the Renilla luciferase gene served as the internal control. Each construct was cotransfected with HIC2 or pcDNA3.1 vector into LN229 glioma cells. The dual‐luciferase reporter assay was performed according to the manufacturer's instructions (Promega, Madison, WI, USA). For the ChIP assay, an antibody against HIC2 (NBP3‐13891, Novus Biological) was used to pull down the DNA that interacted with HIC2. The precipitated DNA samples were further assessed by qRT–PCR. The primers were shown in Appendix S1.

### 2.11 Methylation‐specific PCR (MSP) and bisulfite sequencing PCR (BSP)

Purified gDNA was extracted from six glioma cell lines and one normal immortalized astrocyte using the TIANamp Genomic DNA Kit (TIANGEN Biotech (Beijing) Co., Ltd., Cat. No. 4992199). DNA bisulfite conversion was performed for DNA methylation assessment. After bisulfite treatment, unmethylated “C” was converted into “U”. Using the Methylation‐specific PCR (MSP) Kit (TIANGEN Biotech (Beijing) Co., Ltd., Cat. No. 4992759) and bisulfite‐treated genomic DNA as the template, 400‐bp fragments were amplified in a reaction system of 20 μl. Afterward, PCR cycles were set up for MSP. The sequences of the two primer pairs are shown in Appendix S1. After PCR amplification was complete, 10 μl of reaction products was loaded onto an agarose gel for detection. The bisulfite‐treated DNA was amplified using the methylation‐specific primer set found at http://www.urogene.org/cgi‐bin/methprimer2/MethPrimer.cgi. MSP primers were tested previously to ensure they did not amply DNA that was not bisulfited, and the MSP products of several cell lines were confirmed by direct sequencing, indicating that our MSP system was specific. For BGS, bisulfite‐treated DNA was amplified, and the PCR products were cloned into the pCR4‐Topo vector (Invitrogen). In total, 8–10 colonies were randomly chosen and sequenced.

### 2.12 Statistical analysis

GraphPad Prism 8 software (Version 8.0.0) and R software (Version 4.1.0) were used for statistical analysis and to generate figures. Data distributions were tested using frequency distribution histogram. Differences were analyzed using Student's *t* test for two groups and one‐way ANOVA for multiple groups, while data that do not exhibit a normal distribution were analyzed via a non‐parametric equivalent. *p* < 0.05 was considered statistically significant. *p < 0.05; **p < 0.01; ***p < 0.001.

## 3 RESULTS

### 3.1 HIC2 is hypermethylated in glioma

**FIGURE 1:**
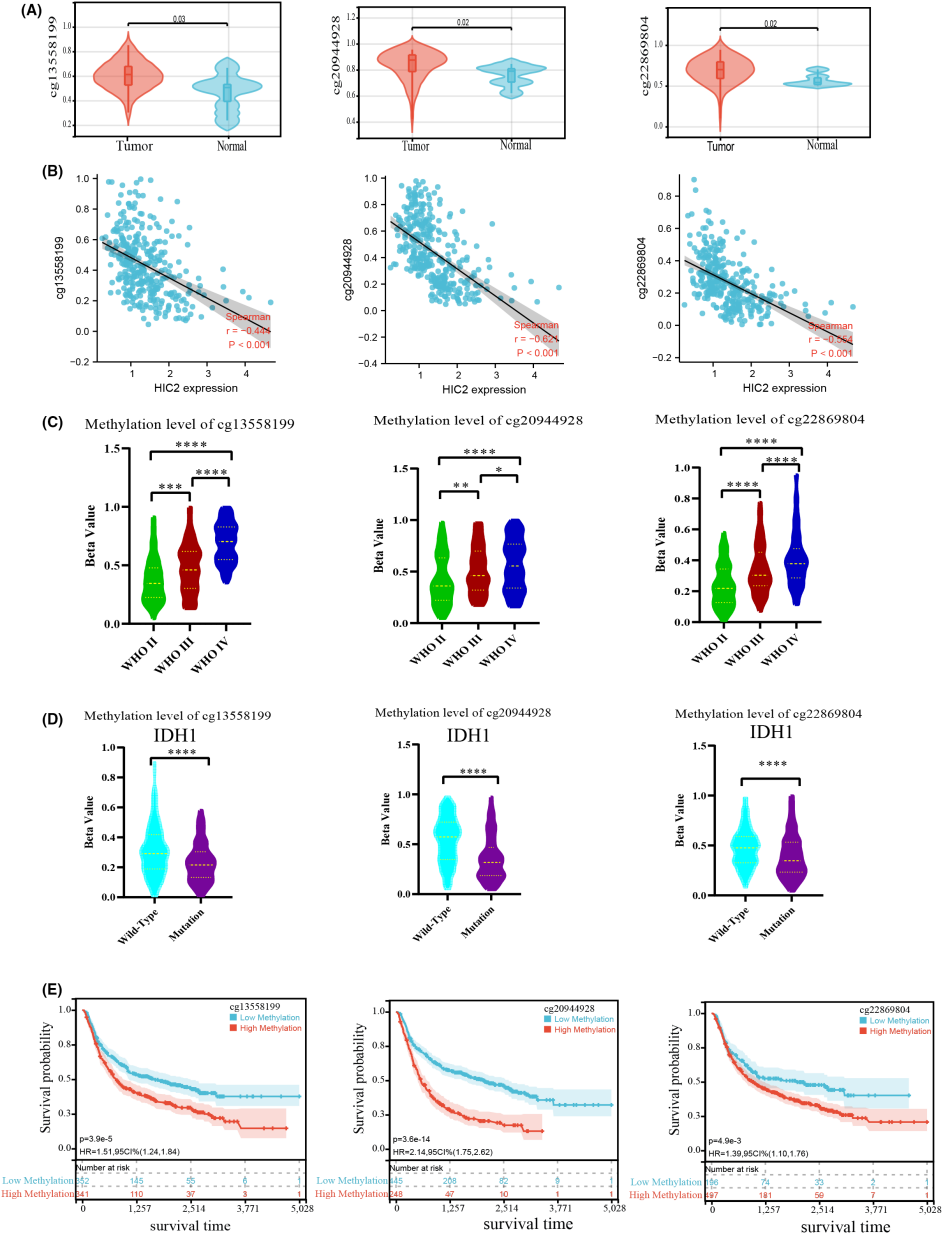
HIC2 is hypermethylated in glioma. (A) HIC2 is highly methylated at CpG sites of the methylation probes cg13558199, cg20944928, and cg22869804 in glioma samples. (B) Methylation of cg13558199, cg20944928, and cg22869804 CpG sites is negatively correlated with HIC2 expression. (C) The methylation level of cg13558199, cg20944928, and cg22869804 CpG sites increases with increasing glioma grade. (D) Methylation of cg13558199, cg20944928, and cg22869804 CpG sites was higher in IDH1 wild‐type samples than in IDH1 mutant samples. (E) High methylation levels of cg13558199, cg20944928, and cg22869804 CpG sites predict poor prognosis in glioma patients (*: *p* < 0.05; **: *p* < 0.01; ***: *p* < 0.001 and ****: *p* < 0.0001; N.S.: not significant).

DNA methylation status is an important regulator of gene expression levels. We first assessed the DNA methylation status of the HIC2 promoter, since HIC2 is abnormally expressed in glioma samples. As shown in Figure 1A, the promoter of HIC2 was highly methylated at three CpG sites, including cg13558199 (*p* < 0.05), cg20944928 (*p* < 0.05), and cg22869804 (*p* < 0.05), in glioma samples. The results of correlation analysis revealed a negative correlation between HIC2 expression and DNA methylation at CpG sites cg13558199 (*r* = −0.444, *p* < 0.001), cg20944928 (*r* = −0.621, *p* < 0.001), and cg22869804 (*r* = −0.554, *p* < 0.001) (Figure 1B). We further investigated the association between HIC2 DNA methylation and clinicopathologic features of glioma. The results showed that DNA methylation of HIC2 at three CpG sites was significantly increased with increasing glioma grade from grade II to grade IV (Figure 1C). In addition, DNA methylation levels of HIC2 were higher in IDH1 wild‐type glioma patients than in IDH1 mutation glioma patients (Figure 1D, *p* < 0.0001). We also analyzed the relationship between 1p/19q codeletion or MGMT promoter methylation and DNA methylation levels of HIC2 at three CpG sites. Results showed that the difference of DNA methylation levels of HIC2 at three CpG sites (cg13558199, cg20944028, and cg22869804) between 1p/19q codel group and 1p/19q non‐codel group was not significance (Figure S1A for TCGA database; Figure S1B for CGGA database). DNA methylation levels of HIC2 at three CpG sites were not different between MGMT promoter methylation group and MGMT promoter un‐methylated group (Figure S1C). Moreover, to investigate the prognostic significance of DNA methylation of HIC2 in glioma patients, survival analysis was performed, and we found that high DNA methylation levels of HIC2 at three CpG sites predicted poor prognosis in glioma patients, implying a tumor suppressor role of HIC2 (Figure 1E). The gene location and primer sites for bisulfite genomic sequencing (BGS) sequence of HIC2 were visualized using the UCSC database and Methprimer database (Figure S2A,B). MSP assay result showed that expression of HIC2 in glioma cells was lower compared to normal immortalized astrocyte, while methylation level of HIC2 in glioma cells was reverse (Figure S2C). We further performed BGS to detect the methylation of individual CpG sites on the HIC2 promoter of four glioma cell lines, and the results showed that HIC2 was highly methylated in four glioma cell lines (Figure S2D). Collectively, our results demonstrated that HIC2 is highly methylated in glioma and that high DNA methylation levels of HIC2 at three CpG sites are associated with poor prognosis.

### 3.2 HIC2 is downregulated in glioma and associated with poor prognosis

**FIGURE 2:**
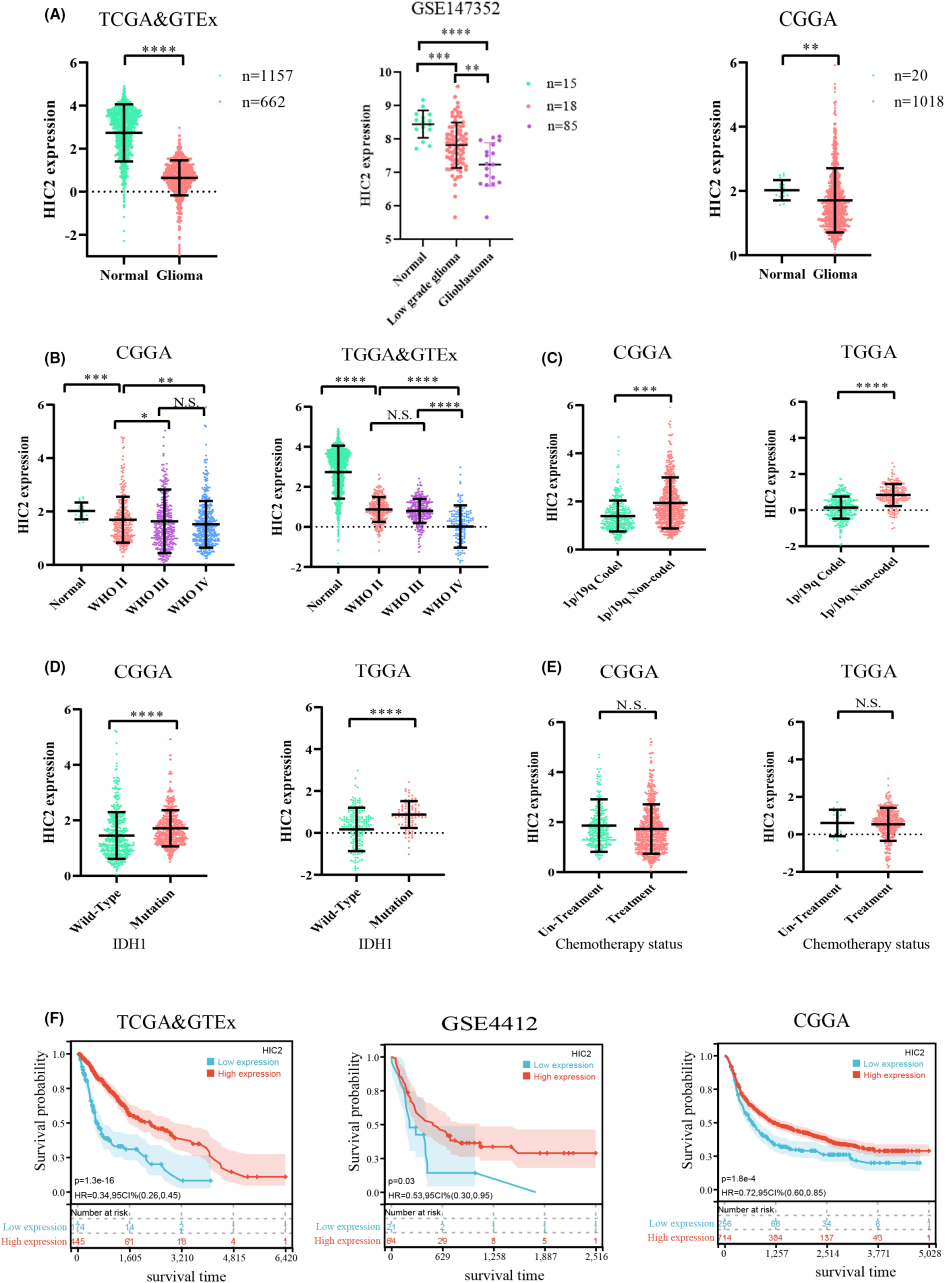
HIC2 is downregulated in glioma and is associated with poor prognosis. (A) Expression of HIC2 is lower in glioma samples than in normal samples based on four databases (left: TCGA and GTEx; middle: GSE147352; right: CGGA). (B) Expression of HIC2 decreases gradually with increasing glioma grade. (C) HIC2 expression is higher in 1p/19q noncodeletion samples than in 1p/19q codeletion samples. (D) HIC2 expression is higher in IDH1 mutant samples than in IDH1 wild‐type samples. (E) HIC2 expression is not different between the TMZ treatment and the non‐TMZ treatment groups. (F) Low expression of HIC2 predicts poor prognosis (*: *p* < 0.05; **: *p* < 0.01; ***: *p* < 0.001 and ****: *p* < 0.0001; N.S.: not significant).

To investigate expression levels of HIC2 in glioma samples, we first downloaded the expression profile of different grades of glioma from four databases: TCGA, GTEx, CGGA, and GEO. A total of 1819 samples in TCGA and GTEx, 118 samples in GSE147352, and 1218 samples in CGGA were included and analyzed in our study. Expression levels of HIC2 were significantly downregulated in glioma samples compared to control samples, as confirmed by TCGA and GTEx (*p* < 0.001), GEO (*p* < 0.001), and CGGA (*p* < 0.01) databases (Figure 2A). Next, expression levels of HIC2 and clinicopathologic features of glioma were analyzed. The results showed that expression levels of HIC2 were gradually decreased with increasing tumor grade from normal to grade IV in the TCGA, GTEx, and CGGA databases (Figure 2B). As a 1p/19q codeletion, MGMT promoter methylation and IDH1 mutation is an important marker for the prognostic evaluation of glioma patients, and we investigated expression of HIC2 and this clinicopathologic feature. HIC2 expression levels were lower in 1p/19q codeletion patients than in 1p/19q noncodeletion patients (Figure 2C). In addition, HIC2 expression levels were lower in IDH1 wild‐type patients than in IDH1 mutation patients (Figure 2D). HIC2 expression is not different between MGMT promoter methylation group and MGMT promoter un‐methylation groups (Figure S3) and as shown in Figure 2E, HIC2 expression did not change after glioma patients were treated with temozolomide. Furthermore, low expression of HIC2 was closely associated with poor OS of glioma patients in TCGA database, in accordance with the data from GSE4412 and CGGA (Figure 2F). Therefore, these results confirmed the low expression of HIC2 in glioma patients, which predicted poor prognosis.

### 3.3 HIC2 is an independent prognostic factor for glioma patients

**FIGURE 3:**
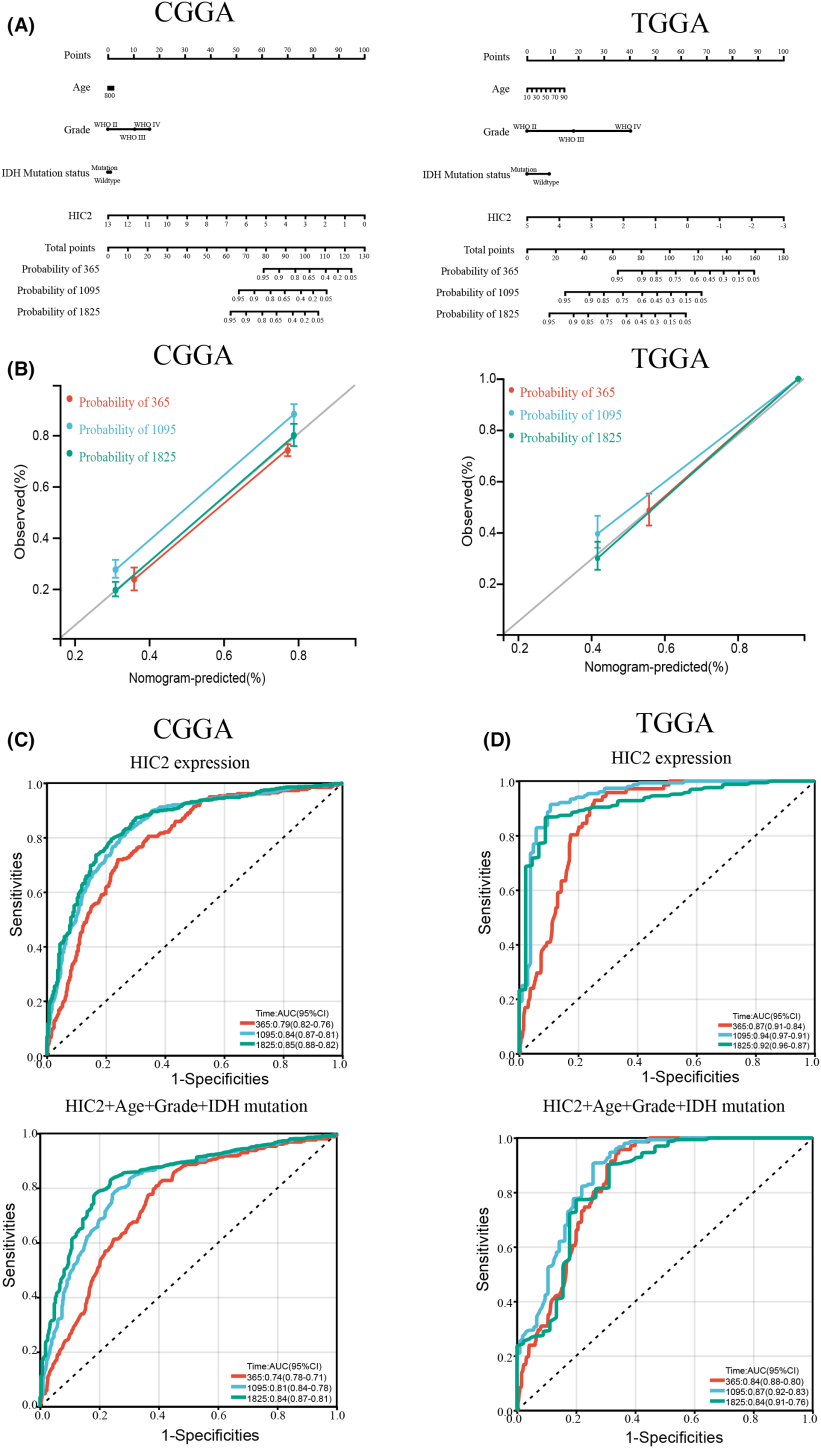
HIC2 is an independent predictive factor for poor prognosis in glioma patients. (A) A nomogram was constructed by integrating HIC2 expression, age, sex, grade, and IDH mutation status based on the CGGA (left) and TCGA (right) datasets. (B) The calibration plot of the nomogram for overall survival prediction at 1‐year (red), 3‐years (blue) and 5‐years (green) in the CGGA (left) and TCGA (right) databases. (C, D) Time‐dependent ROC curve analysis for overall survival prediction at 1‐year (red), 3‐years (blue), and 5‐years (green) in the CGGA (C) and TCGA (D) databases.

Because glioma patients with low HIC2 expression have poor prognosis, we next performed univariate and multivariate Cox regression analyses using data from TCGA and CGGA to explore whether HIC2 is an independent prognostic factor in glioma patients. The results of univariate and multivariate Cox regression analyses showed that IDH1 mutation (univariate HR: 0.39, *p* = 5.1e‐30; multivariate HR: 0.79, *p* = 0.02) and HIC2 (univariate HR: 0.24, *p* = 8.5e‐26; multivariate HR: 0.36, *p* = 7.4e‐13) were independent protective factors for glioma, while age (univariate HR: 1.7, *p* = 1.6e‐09; multivariate HR: 1.26, *p* = 0.01) and grade (univariate HR: 2.6, *p* = 6.4e‐63; multivariate HR: 2.17, *p* = 1.53e‐34) were risk factors for glioma (Figure S4A). Similar results were also obtained from data based on TCGA (Figure S4B). Furthermore, we analyzed the relationship between the risk score and patient follow‐up time, gene expression and clinicopathologic features. As shown in Figure S4C, with the increase in risk score, the survival rate of glioma patients significantly decreased, consistent with the results that HIC2 and IDH1 mutations were protective factors, while age and grade were risk factors in glioma. Next, a nomogram based on these independent prognostic factors was constructed to predict 1‐, 3‐, and 5‐year survival in glioma patients (Figure 3A). The calibrated plot of survival probability shows a high consistency of probability of nomogram survival prediction and the ideal reference line for the CGGA and TCGA databases, and the C‐index of the model was 0.77 (95% CI: 0.75–0.79, *p* = 1.55e‐190) in the CGGA dataset and 0.87 (95% CI: 0.85–0.89, *p* = 1.83–257) in the TCGA dataset (Figure 3B). To investigate the prognostic ability of HIC2 expression in predicting the survival of glioma patients, a ROC curve was constructed. The results showed that HIC2 had high prediction ability for glioma patient overall survival (AUC: 0.79, in 1‐year survival; AUC: 0.84, in 3‐year survival; AUC: 0.85, in 5‐year survival) based on the CGGA dataset, while in TCGA dataset, the AUC of the ROC curve was 0.87 in 1‐year survival, 0.94 in 3‐year survival, and 0.92 in 5‐year survival. Because age, grade, and IDH mutation were enrolled in our Cox regression model as independent prognostic factors, we further constructed the ROC curve by combining HIC2 expression and these parameters. The results showed that the AUC of the ROC curve was 0.74 for 1‐year survival, 0.81 for 3‐year survival, and 0.842 for 5‐year survival based on the CGGA dataset and 0.84 for 1‐year survival, 0.87 for 3‐year survival, and 0.84 for 5‐year survival in the TCGA dataset (Figure 3C,D). Collectively, these findings indicate that HIC2 is an independent prognostic factor in glioma patients and has high prognostic prediction ability.

### 3.4 Enrichment analysis of HIC2 in gliomas

**FIGURE 4:**
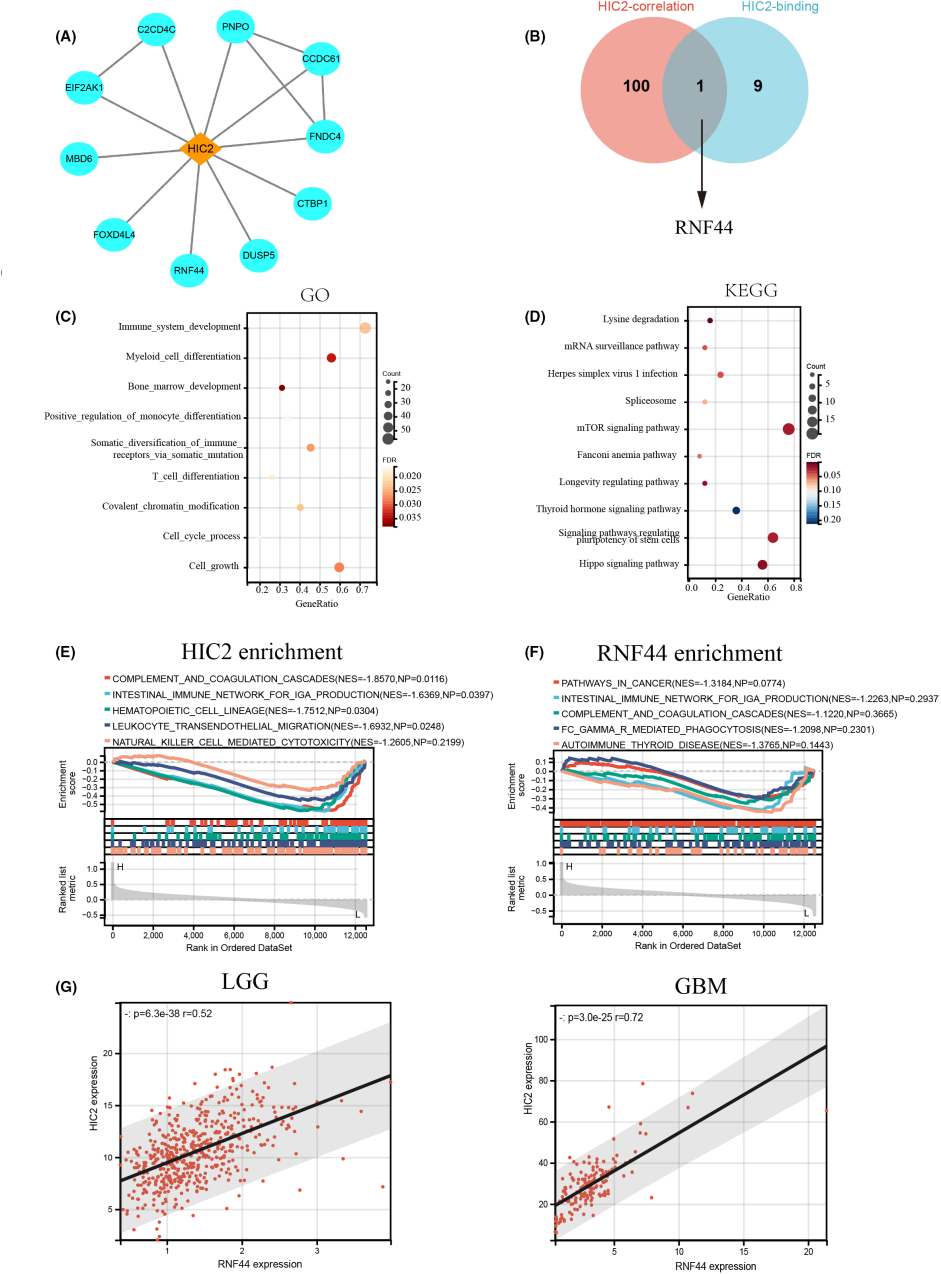
Enrichment analysis of HIC2 in gliomas. (A) Protein–protein interaction network of HIC2 based on predictions from the STRING database. (B) Venn diagram of HIC2‐correlated genes and HIC2‐binding genes. RNF44 was the only gene that was present in both gene sets. (C) GO analysis based on HIC2 expression in glioma. (D) KEGG analysis based on HIC2 expression in glioma. (E) GSEA based on HIC2 expression in glioma. (F) GSEA based on RNF44 expression in glioma. (G) HIC2 expression is positively correlated with RNF44 expression in LGG (left) and GBM (right).

A recent study reported that HIC2 is a transcriptional suppressor, and we suspected that HIC2 could bind to proteins as part of a transcriptional complex. We first performed protein–protein interaction (PPI) analysis of HIC2 using the STRING database, and the interaction network containing 11 nodes was visualized using Cytoscape software (Figure 4A). Then, the top 100 genes correlated with HIC2 expression were identified using the GEPIA2 database, and the intersection of the two gene sets was visualized in a Venn diagram. The results revealed that RNF44 was the only member that occurred in both gene sets, indicating an important function of HIC2 interacting with RNF44 (Figure 4B). Because HIC2 was expressed at low levels in glioma samples, we further investigated the biological function of HIC2 in glioma based on HIC2 expression using GO and KEGG enrichment analyses. The results of GO analysis revealed that a variety of biological functions were associated with HIC2 expression, such as cell growth and immune system development, (Figure 4C). Meanwhile, enrichment analysis of HIC2 expression suggested that the mTOR signaling pathway was potential HIC2‐mediated pathways in glioma cells (Figure 4D). Furthermore, we performed GSEA enrichment analysis based on the expression of HIC2 or RNF44 and found that some immune‐related biological processes were enriched in HIC2‐mediated and RNF44‐mediated processes (Figure 4E,F). The relationship between HIC2 and RNF44 was analyzed by expression level, and the results showed that the expression of HIC2 was positively correlated with RNF44 expression in the LGG (*r* = 0.52, *p* = 6.3e‐38) and GBM datasets (*r* = 0.72, *p* = 3.0e‐25) (Figure 4G). Collectively, our results indicate that HIC2 binds to RNF44 and might act as a transcriptional suppressor in mediating cell growth, immune‐related biological processes, and function through the mTOR signaling pathway.

### 3.5 HIC2 inhibits glioma cell proliferation

**FIGURE 5:**
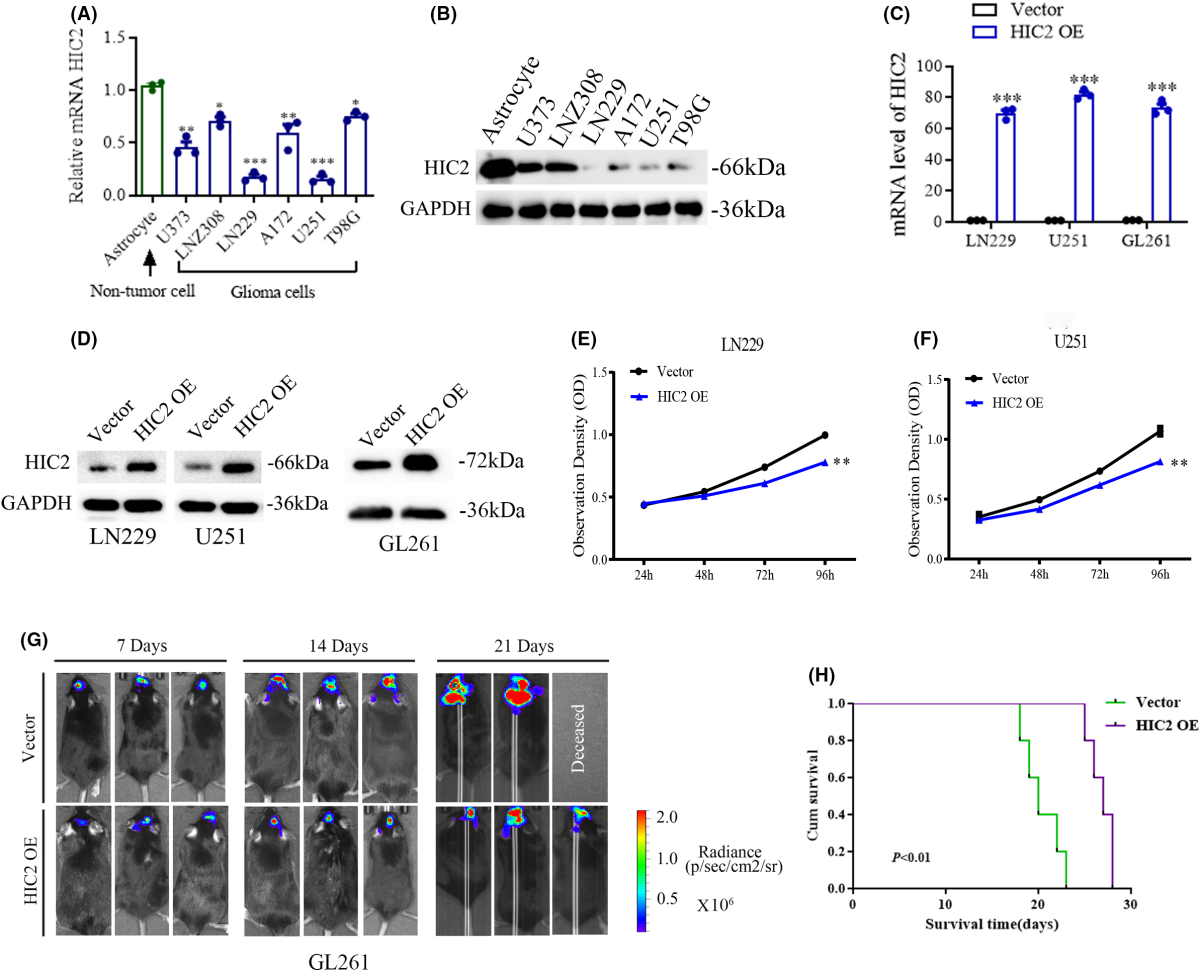
HIC2 impairs glioma cell proliferation. (A, B) mRNA levels of HIC2 in six glioma cells and one human immortalized astrocyte cell, detected using qRT–PCR and western‐blot. (C, D) HIC2 is overexpressed in LN229, U251 and GL261 glioma cells. (E, F) Overexpression of HIC2 impairs cell growth in LN229 and U251 glioma cells. (G) Representative bioluminescence imaging (BLI) images of GL261 models detect on day 7, 14 and 21, respectively. (H) Overall‐survival time of GL261‐bearing C57BL/6 mice in control groups and HIC2 OE group. (*: *p* < 0.05; **: *p* < 0.01; ***: *p* < 0.001 and ****: *p* < 0.0001; N.S.: not significant).

To further investigate the function of HIC2 in glioma cells, we first assessed the expression of HIC2 in six glioma cell lines and one nontumor immortalized astrocyte cell line. As shown in Figure 5A,B, we found that HIC2 was expressed at lower levels in glioma cells than that in the immortalized astrocyte cells, consistent with the results that HIC2 is expressed at low levels in glioma samples. Based on the expression levels of HIC2 in glioma cells, HIC2 was stably overexpressed in LN229, U251, and GL261 glioma cells (Figure 5C,D). The results of the proliferation assay showed that upregulating HIC2 impaired cell growth in both LN229 and U251 glioma cells (Figure 5E,F). We further investigated the growth inhibition role of HIC2 in an orthotopic glioblastoma‐bearing mice by using GL261 cell line in C57BL/6 mice. The results showed that overexpression of HIC2 significantly impaired growth of GL261 glioma cells in vivo (Figure 5G,H). Therefore, our findings confirmed the tumor suppressor role of HIC2 in glioma.

### 3.6 HIC2 interacts with RNF44 and downregulates PTPRN2 expression by binding to the PTPRN2 promoter

**FIGURE 6:**
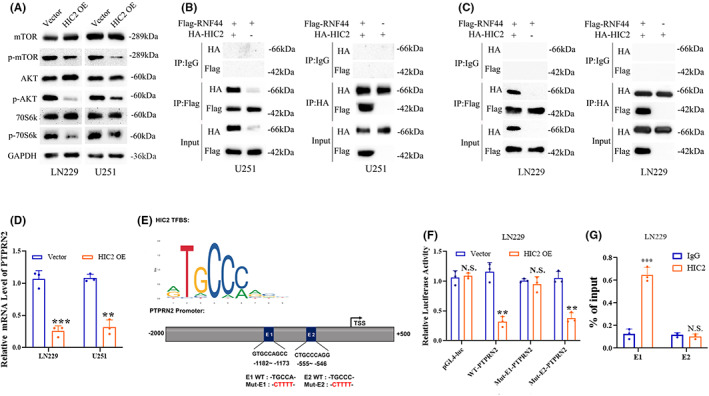
HIC2 interacts with RNF44 and downregulates PTPRN2 expression by binding to the PTPRN2 promoter. (A) Overexpression of HIC2 decreases the phosphorylation of Akt, mTOR, and p70D6k. (B, C) Co‐IP assay show that the HIC2 protein interacts with RNF44 (left: U251; right: LN229). (D) Overexpression of HIC2 decreases mRNA level of PTPRN2. (E) The DNA binding motif of HIC2 and a model describing the binding sites between HIC2 and the promoter of PTPRN2 and the establishment of the mutant E (Mut‐E)‐PTPRN2 promoter constructs. (F) A dual‐luciferase reporter assay showing the relative luciferase activities in LN229 and U251 glioma cells after cotransfection of vector or HIC2 with the Mut‐E1‐PTPRN2 promoter, Mut‐E2‐PTPRN2 promoter, WT‐PTPRN2 promoter or empty construct pGL4‐luc. (G) CHIP assay showing that HIC2 binds to the E1 region of the PTPRN2 promoter.

Since the KEGG analysis suggested that HIC2 might regulate the Akt/mTOR signaling pathway, we assessed phosphorylation levels of key proteins in the Akt/mTOR signaling pathway. As shown in Figure 6A, overexpression of HIC2 decreased the phosphorylation levels of mTOR, AKT and 70S6k in LN229 and U251 glioma cells. HIC2 was suggested to bind to RNF44 in our study by bioinformatics analysis. Therefore, we further performed a Co‐IP assay by cotransfecting a plasmid containing RNF44 with a Flag tag or HIC2 with an HA tag into glioma cells to evaluate the interaction between HIC2 and RNF44. We found that HIC2 physically bound to RNF44 (Figure 6B,C). In addition, we predicted the DNA binding motif and target genes of HIC2 using JASPER (https://jaspar.genereg.net/) and the cistrome database (http://cistrome.org/db/), the target genes of HIC2 were shown in Table 1, which showed that Protein tyrosine phosphatase receptor type N2 (PTPRN2) was the most likely target gene (markered in Bold). The results of the qRT–PCR assay revealed that overexpression of HIC2 downregulated PTPRN2 mRNA levels (Figure 6D). To further verify whether PTPRN2 is a direct target of HIC2, we generated three constructs: the wild‐type (WT)‐PTPRN2 promoter, mutant E1 (Mut‐E1)‐PTPRN2 promoter, and mutant E2 (Mut‐E2)‐PTPRN2 promoter constructs. A model describing the interaction between HIC2 and the promoter region (E1 and E2) of PTPRN2 and the establishment of the Mut‐E1‐PTPRN2 promoter, Mut‐E2‐PTPRN2 promoter, and WT‐PTPRN2 promoter is shown in Figure 6E. The empty luciferase reporter construct (pGL4‐luc) served as a control. The relative luciferase activity in LN229 glioma cells was markedly decreased after cotransfection of HIC2 and WT‐PTPRN2‐luc or HIC2 and Mut‐E2‐luc, while luciferase activity was not affected after cotransfection of HIC2 and Mut‐E1‐luc (Figure 6F). Furthermore, a ChIP assay was performed to verify the binding of HIC2 to the PTPRN2 promoter. The qRT–PCR results showed that in the chromatin fraction pulled down by the anti‐HIC2 antibody, only the E1 region of the PTPRN2 promoter was detected (Figure 6G). Collectively, our findings suggest that HIC2 interacts with RNF44 and downregulates PTPRN2 expression by binding to its promoter.

**TABLE 1:** Target genes of HIC2 predicted by cistrome database

Gene	Score	Coordinate
**PTPRN2**	0.599	chr7:157539055–158,587,822
LOC105378947	0.482	chr1:586286–611,296
ARHGEF18	0.462	chr19:7439687–7,472,477
PEX11G	0.071	chr19:7476874–7,489,034
OR4F16	0.046	chr1:685715–686,653
TEX45	0.039	chr19:7497547–7,508,449
FRG2B	0.013	chr10:133623894–133,626,794
ZNF358	0.011	chr19:7515285–7,521,024
MCOLN1	0.007	chr19:7522623–7,534,008
NCAPG2	0.007	chr7:158631308–158,667,237
PNPLA6	0.003	chr19:7534163–7,561,763
BAGE2	0.002	chr21:10413531–10,491,614
EFHC2	0.002	chrX:44147871–44,343,671
DEFB115	0.001	chr20:31257663–31,259,631
LOC107984841	0.001	chr1:733139–744,656

### 3.7 HIC2 correlated with immune cell infiltration in glioma

Because HIC2 is involved in immunomodulatory signaling pathways in gliomas, we further investigated the correlation between HIC2 expression and immune cell infiltration. Firstly, we analyze HIC2 expression and six types of immune cell infiltration in the LGG and GBM datasets. The results revealed that HIC2 was significantly negatively correlated with the infiltration of M2 macrophages but positively correlated with neutrophils and NK cells in both LGG and GBM (Figure S5A). We found that HIC2 expression was significantly negatively correlated with M2 macrophages and CD8^+^ T cells but positively correlated with memory B cells, M1 macrophages, neutrophils, and NK cells based on the data from CGGA and TCGA (Figure S5B). In addition, DNA methylation of the HIC2 gene at cg13558199, cg20944928 and cg22869804 sites was positively correlated with M2 macrophages and CD8+ T cells but negatively correlated with M1 macrophages, neutrophils and NK cells (Figure S5C). High expression of HIC2 was accompanied by low immune scores (*p* < 0.0001, Figure S5D). Furthermore, we found that 5 immune inhibitors (KDR, TIGIT, VTCN1, LAG3 and ADORA2A) and 2 immune stimulators (ICOS and TNFRSF25) were significantly associated with HIC2 in gliomas (Figure S5E). The correlations between HIC2 expression and immune checkpoints and classical phenotypes of macrophages and neutrophils were also analyzed. We found that HIC2 was positively associated with immune checkpoints, such as PDL1, PDL2, LAG3, and CTLA4. In addition, HIC2 was negatively correlated with M2 markers of tumor‐associated macrophages (TAMs) and N2 phenotype markers of tumor‐associated neutrophils (TANs) but positively correlated with M1 markers of TAMs and NI1 markers of TANs (Figure S5F‐H). Collectively, our results suggest that HIC2 expression might be involved in the immune response.

## 4 DISCUSSION

Gliomas are a heterogeneous group of intracranial neoplasms of glial origin. They are the most common type of primary tumor (40%) in the central nervous system. A growing number of epigenetic prognostic markers, such as ARF, CDKN2B, RB1, APC, CDH1, ESR1, GSTP1, MYOD1, and HIC1, are frequently being discovered in gliomas.^14^, ^15^ In gliomas, the methylation status of the promoter for MGMT has been verified to be associated with glioma progression and has been identified as a biomarker to predict whether glioma patients will benefit from TMZ treatment.^16^ HIC1 is a novel tumor suppressor gene that acts as a negative transcriptional regulator and growth suppressor and is commonly found to be methylated in many human cancers, including glioma.^17^, ^18^ HIC2 is a human homolog of the putative tumor suppressor gene HIC1.^7^ The structural and biochemical characteristics and functions of HIC2 in tumorigenesis a transcription factor remain unknown.

We were curious whether HIC2 plays the same role as HIC1 in glioma. In fact, we found that HIC2 is downregulated in glioma cell lines compared to nontumor immortalized astrocyte cells, and mRNA expression levels of HIC2 were negatively correlated with DNA hypermethylation in its promoter, which may explain why, at least in part, the low expression of HIC2 in glioma tissues was due to epigenetic silencing. Moreover, further analysis showed that high DNA methylation levels or lower mRNA expression levels of HIC2 were correlated with worse overall survival and more malignant clinicopathological phenotypes. Previous studies reported that 1p/19q codeletion always means better prognosis. In this study, we found that expression of HIC2 was lower in 1p/19q codeletion group than that in 1p/19q non‐codeletion group, which seem to be a controversial result. However, we also found that the difference of DNA methylation levels of HIC2 at three CpG sites (cg13558199, cg20944028 and cg22869804) between 1p/19q codel group and 1p/19q non‐codel group was not significance, which suggested that downregulation of HIC2 in 1p/19q codeletion group might not result from DNA methylation. In our opinion, maybe some complex factors resulted in the controversial result, which need further investigation. ROC analysis further verified that HIC2 represents a sensitive indicator to predict 1‐year, 3‐year, and 5‐year survival rates of patients, indicating the value of HIC2 as a prognostic biomarker for gliomas. Taken together, HIC2 could be used as a potentially independent prognostic biomarker for gliomas.

HIC2 was found to be a pivotal transcriptional activator of SIRT1 and consequently upregulates SIRT1 in the heart and potentially many other SIRT‐regulated physiological processes, which might protect the heart from I/R injury.^19^ However, further experimental data found that HIC2 acts as a tumor suppressor gene and transcription repressor in glioma cells. When expression of HIC2 was upregulated, the proliferation of glioma cells was significantly inhibited both in vitro and in vivo. Glioma microenvironment and tumor metabolism are important factors in tumor progression. However, we only focused on the changes in tumor proliferation ability in vivo experiments. Exploring the changes in glioma microenvironment and tumor metabolism using strengthened MRI experiments may help us better understand the process of tumor microenvironment and guide the clinical treatment of glioma.^20^, ^21^, ^22^

When KEGG and GO enrichment analyses were performed, the major biological functions of HIC2 were clustered into cell growth and mTOR/AKT pathways. Then, we verified that the mTOR/AKT pathway was inactivated after overexpression of HIC2 in immunoblotting experiments.

It has been reported that RNF44 is upregulated in melanoma cells due to hyperactivation of the ERK/AKT pathway.^23^ We found that HIC2 interacts with the RNF44 protein through PPI analysis, and we further verified that the two proteins physically bound using a Co‐IP assay. Further bioinformatics analysis showed that HIC2 and RNF44 might be involved in mTOR/AKT pathways. However, we do not yet know how these two proteins function in the AKT pathway after physical binding, which requires further investigation.

PTPRN2 was identified as a new regulator in the progression of glioma by comprehensive protein tyrosine phosphatase mRNA profiling.^24^ As a transcription factor, numerous target genes were found to be transcriptionally regulated by HIC2 through bioinformatics analysis, and the PTPRN2 gene ranked as the top of these target gene sets. Then, we verified that PTPRN2 was indeed transcriptionally inhibited by direct binding of HIC2 to promotor element 1 (located 1173 bp upstream of the transcription start site). Although we did not further examine the function of the target gene PTPRN2, limited and contradictory literature is currently reported regarding PTPRN2's involvement in cancer. Downregulation of PTPRN2 in metastatic breast cancer cells inhibited migratory potential and proliferation of xenograft tumors in vivo.^25^ However, another study found that PTPRN2 did not correlate with glioma patient survival.^24^ Hypermethylation of the PTPRN2 promoter region in glioblastomas suggests tumor suppressor roles.^26^ The biological impact of PTPRN2 on tumors may thus be context dependent, and the role of PTPRN2 in glioma biology needs further investigation.

The tumor microenvironment consists of stromal cell (fibroblasts), immune cells (infiltrating lymphocytes, Macrophages), and tumor cells and interaction among these cells contributes to the tumor malignant progression.^27^, ^28^ Previous studies suggested neutrophils and macrophages are the main tumor infiltrating cells, which correlated with clinical prognosis of gliomas.^29^, ^30^ In our study, GO and KEGG enrichment analysis implied an immune‐mediated role of HIC2 on glioma progression. We investigated the relationship between HIC2 expression and immune cell infiltration by using ESTIMATE, CIBERSORT, and TIMER algorithms. We found that HIC2 expression correlated with macrophage and neutrophil infiltration, which implied that HIC2 played a potential role in the tumor microenvironment. Furthermore, expression of HIC2 negatively correlated to the markers of M2‐type TAMs and N2 type TANs, implying an anti‐tumor function of HIC2 on glioma progression by mediating macrophage and neutrophil infiltration. However, the detailed molecular mechanisms by which HIC2 plays its role is still unclear.

Previous study reported a 7‐gene panel of promoter methylation which could be helpful in brain tumor diagnosis or characterization.^31^ However, our study only evaluated single gene methylation, which has limited value. In summary, our study revealed that HIC2 is hypermethylated in glioma and that HIC2 is an independent protective factor in glioma, suggesting that HIC2 represents a novel biomarker for the prognosis prediction of gliomas. In addition, mechanistic analysis implied that HIC2 might bind to RNF44 and inhibit PTPRN2 expression.

## AUTHOR CONTRIBUTIONS

HPC design the study and draft revision. FFL and YFL collected the data and performed the analysis. FFL performed the qRT‐PCR, western‐blot assay. YFL, EDC, and YY performed the animal experiment. HPC, FFL, and YFL drafted the manuscript and coordinated data collection. DZ, YY, KT, DXZ, and HPC supplied the funding. All authors read and approved the final manuscript.

## FUNDING INFORMATION

The study design, data collection, data analysis, manuscript preparation, and publication decisions of this work were supported by NSFC Incubation Project of Guangdong Provincial People's Hospital (KY0120220043) and National Natural Science Foundation of China (No. 82203081) to HPC; Natural Science Foundation of Guangdong Province of China (2022A1515012540) to DZ; Guangzhou Municipal Science and Technology Bureau (202002030128) to DXZ; National Natural Science Foundation of China (No. 81901250), High‐level Hospital Construction Project of Guangdong Provincial People's Hospital (No. DFJH201924) and Natural Science Foundation of Guangdong Province (2019A1515010104) to YY; Guangzhou Municipal Science and Technology Bureau (201904010348) to KT.

## CONFLICT OF INTEREST

The authors declare no competing interests.

## Supporting information


Appendix S1

Figure S1
Figure S2
Figure S3
Figure S4
Figure S5

## Data Availability

All the analysis data were accessed from TCGA database (https://portal.gdc.cancer.gov/), CGGA database (https://www.cgga.org.cn/) and GEO database (https://www.ncbi.nlm.nih.gov/geo/).

## References

[1] Gritsch S , Batchelor TT , Gonzalez Castro LN . Diagnostic, therapeutic, and prognostic implications of the 2021 World Health Organization classification of tumors of the central nervous system. Cancer. 2022;128(1):47‐58. 34633681 10.1002/cncr.33918

[2] Komori T . Grading of adult diffuse gliomas according to the 2021 WHO classification of tumors of the central nervous system. Lab Investig. 2022;102(2):126‐133. 10.1038/s41374-021-00667-6 34504304

[3] Wen PY , Weller M , Lee EQ , et al. Glioblastoma in adults: a Society for Neuro‐Oncology (SNO) and European Society of Neuro‐Oncology (EANO) consensus review on current management and future directions. Neuro‐Oncology. 2020;22(8):1073‐1113. 32328653 10.1093/neuonc/noaa106 PMC7594557

[4] Ostrom QT , Cioffi G , Waite K , Kruchko C , Barnholtz‐Sloan JS . CBTRUS statistical report: primary brain and other central nervous system tumors diagnosed in the United States in 2014–2018. Neuro‐Oncology. 2021;23(12 Suppl 2):iii1‐iii105. 34608945 10.1093/neuonc/noab200 PMC8491279

[5] Oldrini B , Vaquero‐Siguero N , Mu Q , et al. MGMT genomic rearrangements contribute to chemotherapy resistance in gliomas. Nat Commun. 2020;11(1):3883. 32753598 10.1038/s41467-020-17717-0 PMC7403430

[6] Franceschi E , Lamberti G , Visani M , et al. Temozolomide rechallenge in recurrent glioblastoma: when is it useful? Future Oncol. 2018;14(11):1063‐1069. 29741106 10.2217/fon-2017-0681

[7] Pinte S , Stankovic‐Valentin N , Deltour S , Rood BR , Guerardel C , Leprince D . The tumor suppressor gene HIC1 (hypermethylated in cancer 1) is a sequence‐specific transcriptional repressor: definition of its consensus binding sequence and analysis of its DNA binding and repressive properties. J Biol Chem. 2004;279(37):38313‐38324. 15231840 10.1074/jbc.M401610200

[8] Costoya JA . Functional analysis of the role of POK transcriptional repressors. Brief Funct Genomic Proteomic. 2007;6(1):8‐18. 17384421 10.1093/bfgp/elm002

[9] Deltour S , Pinte S , Guerardel C , Leprince D . Characterization of HRG22, a human homologue of the putative tumor suppressor gene HIC1. Biochem Biophys Res Commun. 2001;287(2):427‐434. 11554746 10.1006/bbrc.2001.5624

[10] Fleuriel C , Touka M , Boulay G , Guerardel C , Rood BR , Leprince D . HIC1 (hypermethylated in cancer 1) epigenetic silencing in tumors. Int J Biochem Cell Biol. 2009;41(1):26‐33. 18723112 10.1016/j.biocel.2008.05.028 PMC2631403

[11] Fujii H , Biel MA , Zhou W , Weitzman SA , Baylin SB , Gabrielson E . Methylation of the HIC‐1 candidate tumor suppressor gene in human breast cancer. Oncogene. 1998;16(16):2159‐2164. 9572497 10.1038/sj.onc.1201976

[12] Dykes IM , van Bueren KL , Ashmore RJ , et al. HIC2 is a novel dosage‐dependent regulator of cardiac development located within the distal 22q11 deletion syndrome region. Circ Res. 2014;115(1):23‐31. 24748541 10.1161/CIRCRESAHA.115.303300

[13] Dykes IM , van Bueren KL , Scambler PJ . HIC2 regulates isoform switching during maturation of the cardiovascular system. J Mol Cell Cardiol. 2018;114:29‐37. 29061339 10.1016/j.yjmcc.2017.10.007 PMC5807030

[14] Reifenberger G , Wirsching HG , Knobbe‐Thomsen CB , Weller M . Advances in the molecular genetics of gliomas ‐ implications for classification and therapy. Nat Rev Clin Oncol. 2017;14(7):434‐452. 28031556 10.1038/nrclinonc.2016.204

[15] Ehrlich M . DNA hypermethylation in disease: mechanisms and clinical relevance. Epigenetics. 2019;14(12):1141‐1163. 31284823 10.1080/15592294.2019.1638701 PMC6791695

[16] Mathur R , Zhang Y , Grimmer MR , et al. MGMT promoter methylation level in newly diagnosed low‐grade glioma is a predictor of hypermutation at recurrence. Neuro‐Oncology. 2020;22(11):1580‐1590. 32166314 10.1093/neuonc/noaa059 PMC8444710

[17] Kumar S . P53 induction accompanying G2/M arrest upon knockdown of tumor suppressor HIC1 in U87MG glioma cells. Mol Cell Biochem. 2014;395(1–2):281‐290. 24992983 10.1007/s11010-014-2137-9

[18] Parrella P , Scintu M , Prencipe M , et al. HIC1 promoter methylation and 17p13.3 allelic loss in invasive ductal carcinoma of the breast. Cancer Lett. 2005;222(1):75‐81. 15837543 10.1016/j.canlet.2004.08.026

[19] Song JY , Lee SH , Kim MK , et al. HIC2, a new transcription activator of SIRT1. FEBS Lett. 2019;593(14):1763‐1776. 31127867 10.1002/1873-3468.13456

[20] Stadlbauer A , Oberndorfer S , Zimmermann M , et al. Physiologic MR imaging of the tumor microenvironment revealed switching of metabolic phenotype upon recurrence of glioblastoma in humans. J Cereb Blood Flow Metab. 2020;40(3):528‐538. 30732550 10.1177/0271678X19827885 PMC7026844

[21] Zarghami N , Soto MS , Perez‐Balderas F , et al. A novel molecular magnetic resonance imaging agent targeting activated leukocyte cell adhesion molecule as demonstrated in mouse brain metastasis models. J Cereb Blood Flow Metab. 2021;41(7):1592‐1607. 33153376 10.1177/0271678X20968943 PMC8217895

[22] Stokes AM , Bergamino M , Alhilali L , et al. Evaluation of single bolus, dual‐echo dynamic susceptibility contrast MRI protocols in brain tumor patients. J Cereb Blood Flow Metab. 2021;41(12):3378‐3390. 34415211 10.1177/0271678X211039597 PMC8669280

[23] Li YY , Wu C , Shah SS , et al. Degradation of AMPK‐alpha1 sensitizes BRAF inhibitor‐resistant melanoma cells to arginine deprivation. Mol Oncol. 2017;11(12):1806‐1825. 29094484 10.1002/1878-0261.12151 PMC5709618

[24] Bourgonje AM , Verrijp K , Schepens JT , et al. Comprehensive protein tyrosine phosphatase mRNA profiling identifies new regulators in the progression of glioma. Acta Neuropathol Commun. 2016;4(1):96. 27586084 10.1186/s40478-016-0372-x PMC5009684

[25] Sengelaub CA , Navrazhina K , Ross JB , Halberg N , Tavazoie SF . PTPRN2 And PLCbeta1 promote metastatic breast cancer cell migration through PI(4,5)P2‐dependent Actin remodeling. EMBO J. 2016;35(1):62‐76. 26620550 10.15252/embj.201591973 PMC4717998

[26] Lee EJ , Rath P , Liu J , et al. Identification of global DNA methylation signatures in glioblastoma‐derived cancer stem cells. J Genet Genomics. 2015;42(7):355‐371. 26233891 10.1016/j.jgg.2015.06.003 PMC4648292

[27] Bader JE , Voss K , Rathmell JC . Targeting metabolism to improve the tumor microenvironment for cancer immunotherapy. Mol Cell. 2020;78(6):1019‐1033. 32559423 10.1016/j.molcel.2020.05.034 PMC7339967

[28] Alvarez‐Breckenridge C , Markson SC , Stocking JH , et al. Microenvironmental landscape of human melanoma brain metastases in response to immune checkpoint inhibition. Cancer Immunol Res. 2022;10(8):996‐1012. 35706413 10.1158/2326-6066.CIR-21-0870 PMC10201927

[29] Rahbar A , Cederarv M , Wolmer‐Solberg N , et al. Enhanced neutrophil activity is associated with shorter time to tumor progression in glioblastoma patients. Onco Targets Ther. 2016;5(2):e1075693. 10.1080/2162402X.2015.1075693 PMC4801436 27057448

[30] Zha C , Meng X , Li L , et al. Neutrophil extracellular traps mediate the crosstalk between glioma progression and the tumor microenvironment via the HMGB1/RAGE/IL‐8 axis. Cancer Biol Med. 2020;17(1):154‐168. 32296583 10.20892/j.issn.2095-3941.2019.0353 PMC7142852

[31] Majchrzak‐Celinska A , Dybska E , Barciszewska AM . DNA methylation analysis with methylation‐sensitive high‐resolution melting (MS‐HRM) reveals gene panel for glioma characteristics. CNS Neurosci Ther. 2020;26(12):1303‐1314. 32783304 10.1111/cns.13443 PMC7702229

