# Supplementary material for: Hypermethylation of HIC2 is a potential prognostic biomarker and tumor suppressor of glioma based on bioinformatics analysis and experiments
Source: CNS Neurosci Ther. 2023 Jan 17;29(4):1154–67. doi: 10.1111/cns.14093 (PMC10018090; doi:10.1111/cns.14093)

Full unedited gel/blot for Figure 5

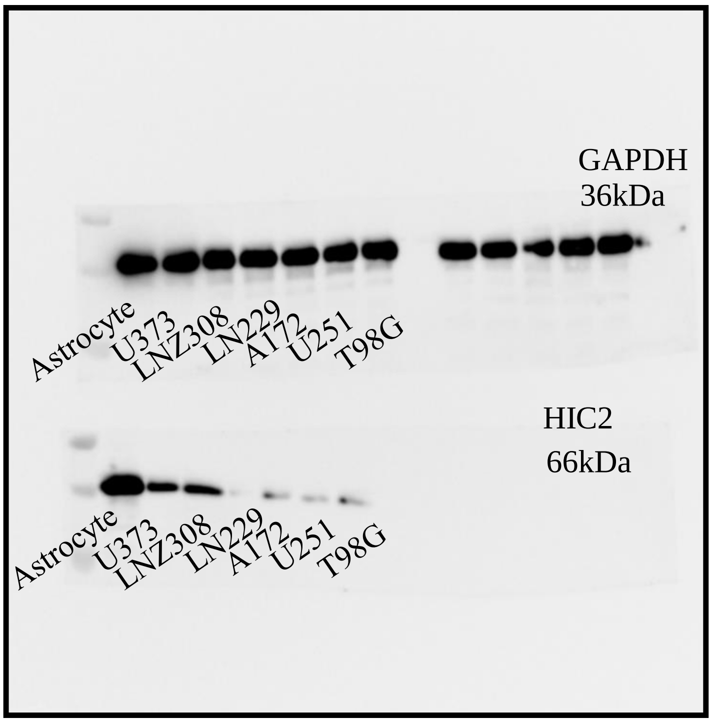

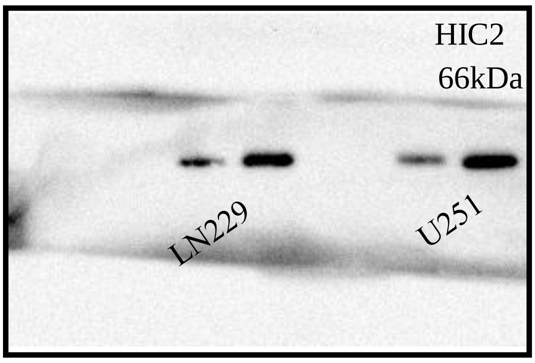

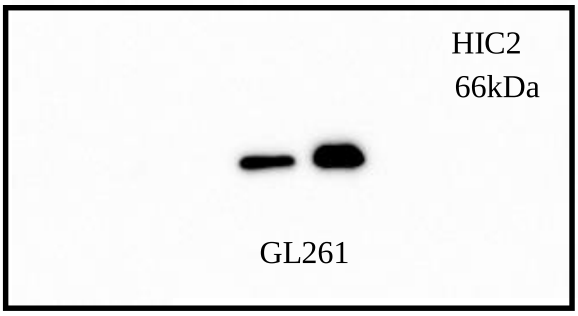

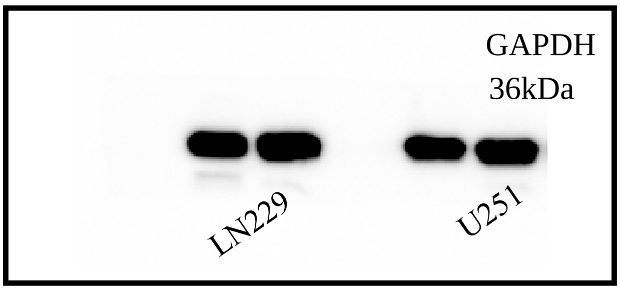

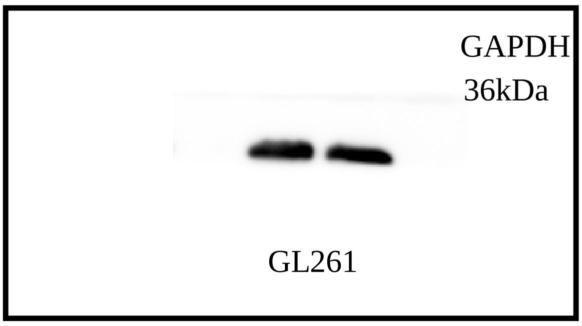

Full unedited gel/blot for Figure 6

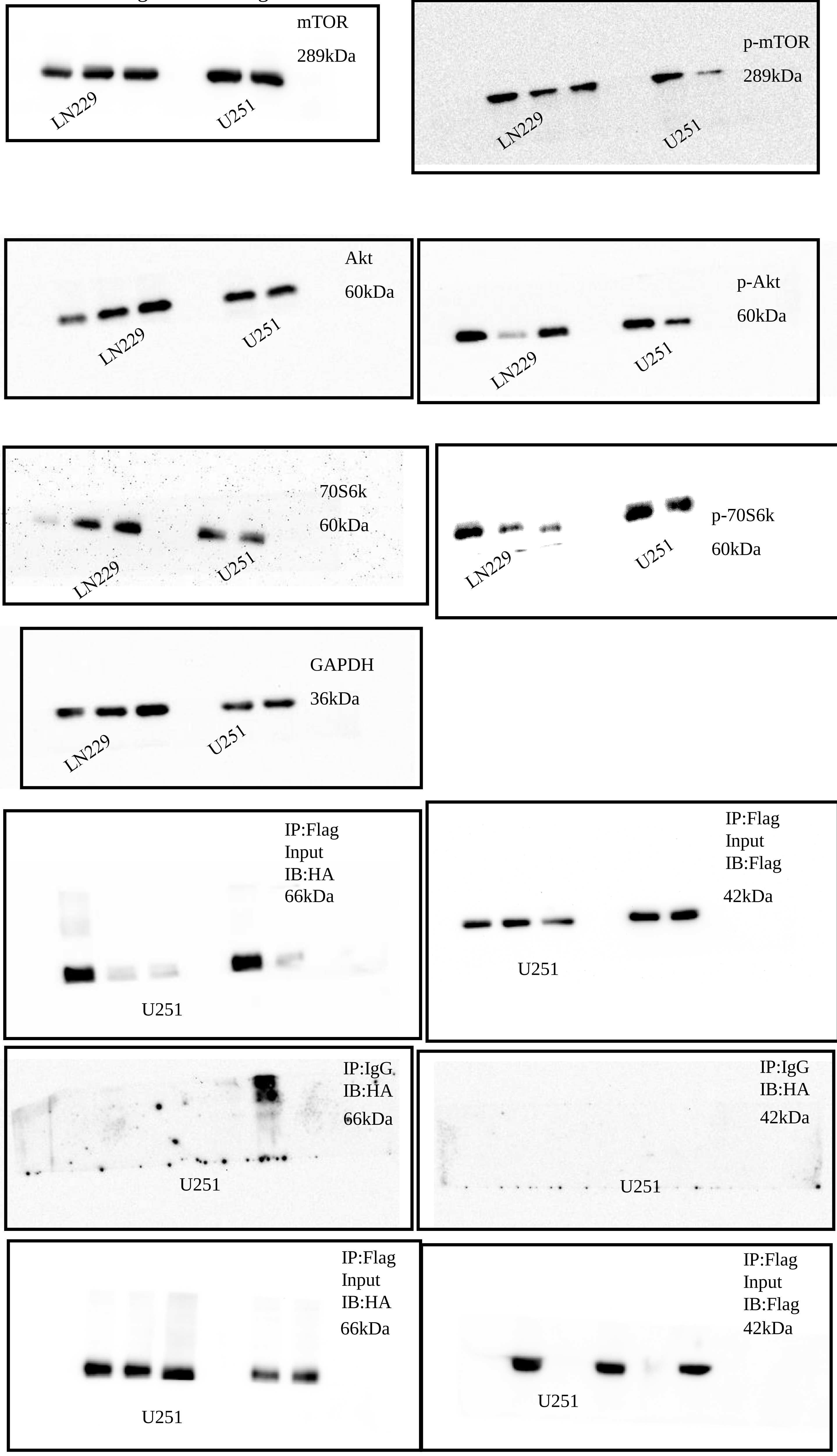

Full unedited gel/blot for Figure 6

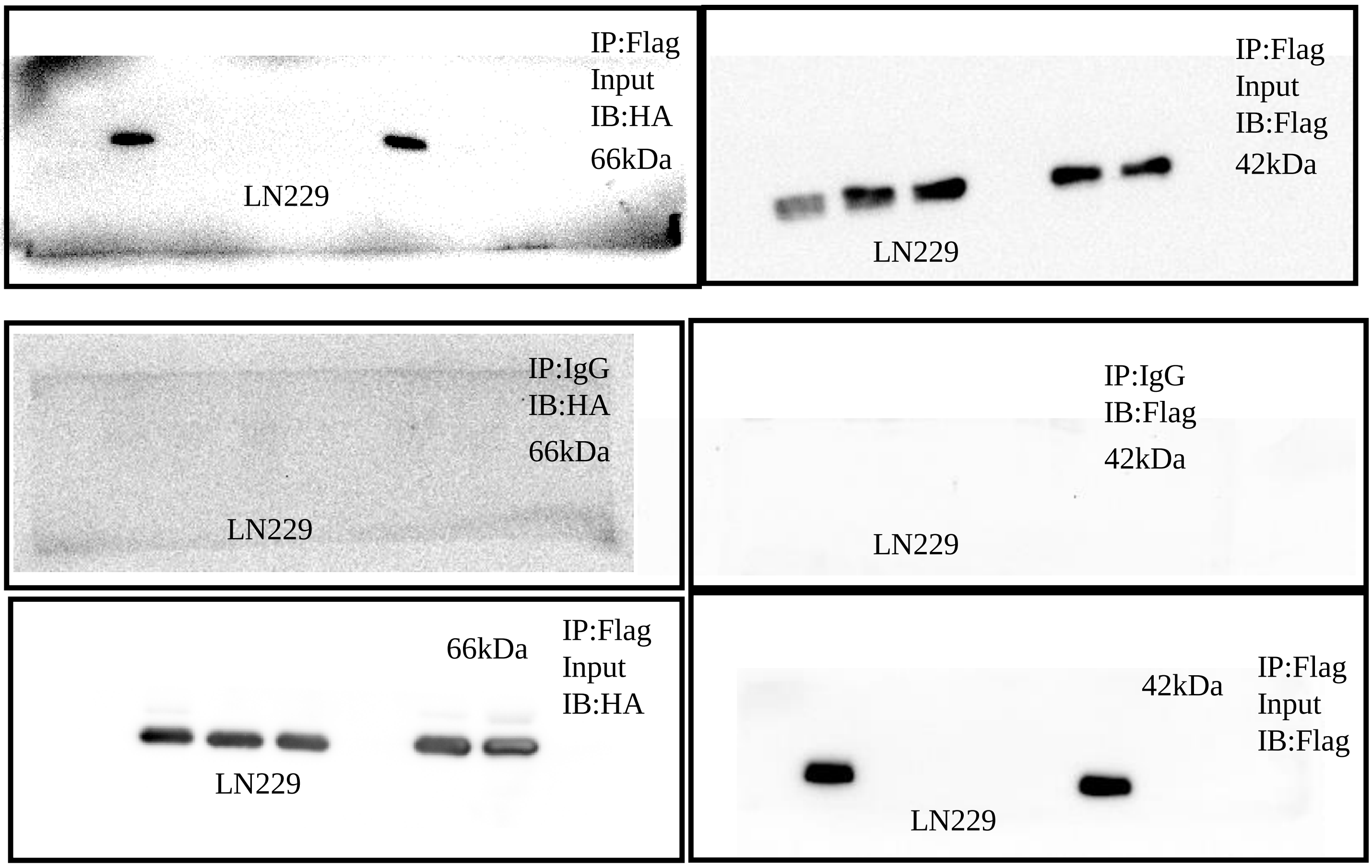

Full unedited gel/blot for supplementary figure 2

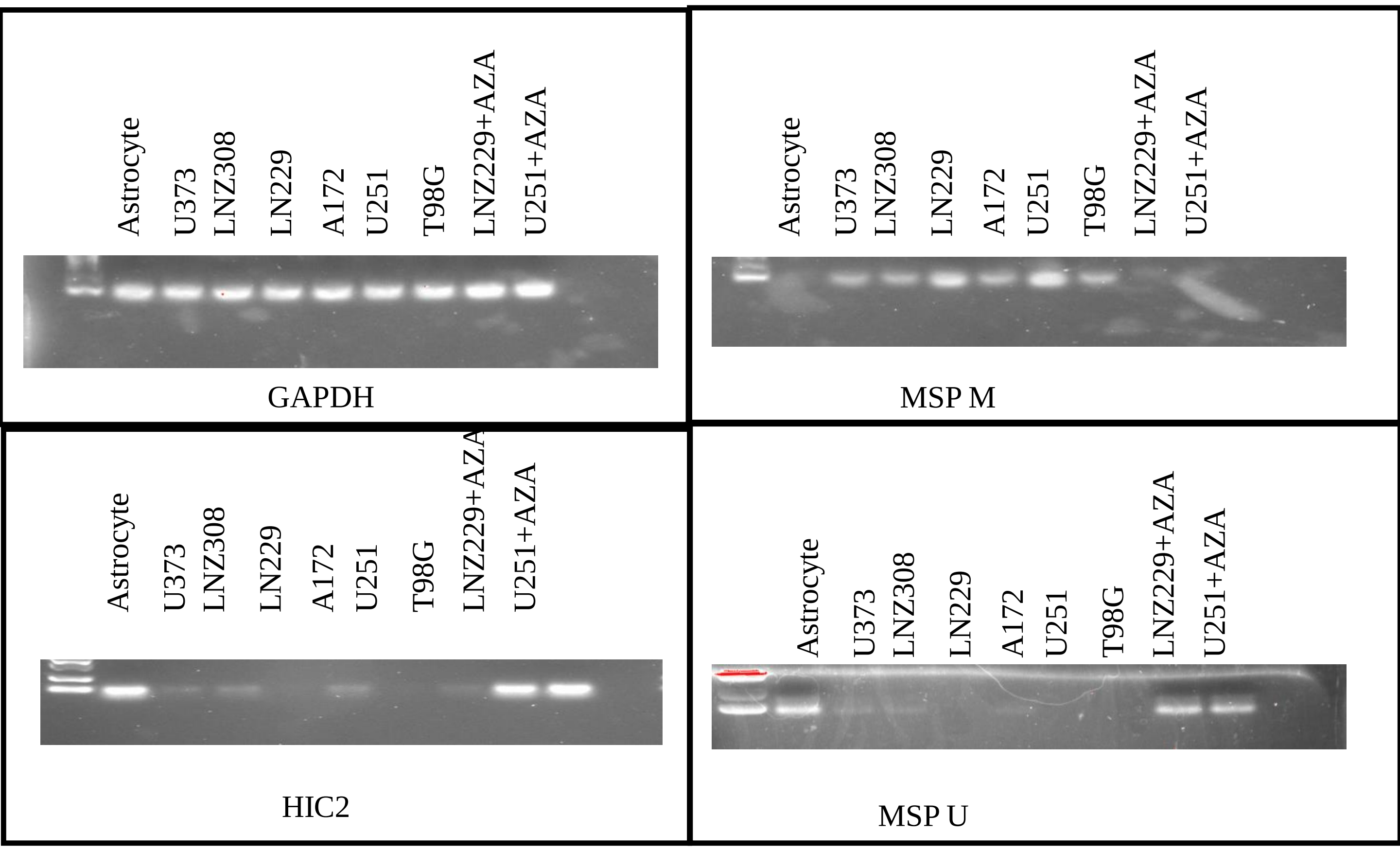

Supplement: Supplementary file 1 — Appendix S1 [file CNS-29-1154-s002.pdf]
