# Supplementary material for: Hypermethylation of HIC2 is a potential prognostic biomarker and tumor suppressor of glioma based on bioinformatics analysis and experiments
Source: CNS Neurosci Ther. 2023 Jan 17;29(4):1154–67. doi: 10.1111/cns.14093 (PMC10018090; doi:10.1111/cns.14093)

Primers were as follow:

Gene	Sequence of primer (5’-3’)
METHYLATION-F	GTCGGGTTGGCGGTAGGCGG
METHYLATION-R	ACCGCCCCTCACCTCGTATA
UNMETHYLATION-F	GGGTGTTGAGGATAAGGGTT
UNMETHYLATION-R	TCTAACCCACAAAACCCAAAC
BGS-F	CCCCCAGCTGCCCCCTGTCC
BGS-R	GGCATCTGGCCCACAAAGCC
hHIC2-F	AGACTCACACGGAGGAAGAGCT
hHIC2-R	GTCTTCTCGCAGACCGAACACT
mHIC2-F	TTCGATGAAATCTACGAGGAGGA
mHIC2-R	GTCCAAATACGGTGACGTGAC

ChIP primers for PTPRN2 promoter:

Gene	Sequence of primer (5’-3’)
E1–F	ATTGCAGATGTTTGGGCTAC
E1–R	TGCCTGGCCACCAGGGCCAT
E2–F	ACGTTTAAATTAAAGAAACG
E2–R	GCGCCCTTGGCCCGGACGGG

Supplementary figure 1. Relationship between 1p/19q codeletion, MGMT promoter methylation and DNA methylation levels of HIC2.

A: Methylation of cg13558199, cg20944928 and cg22869804 CpG sites is not different between 1p/19q Codel group and 1p/19q Non-codel group according to the TCGA database. **B, C:** Methylation of cg13558199, cg20944928 and cg22869804 CpG sites is not different between 1p/19q Codel (MGMT promoter methylation) group and 1p/19q Non-codel (MGMT promoter un-methylation) group according to the CGGA database.

Supplementary figure 1

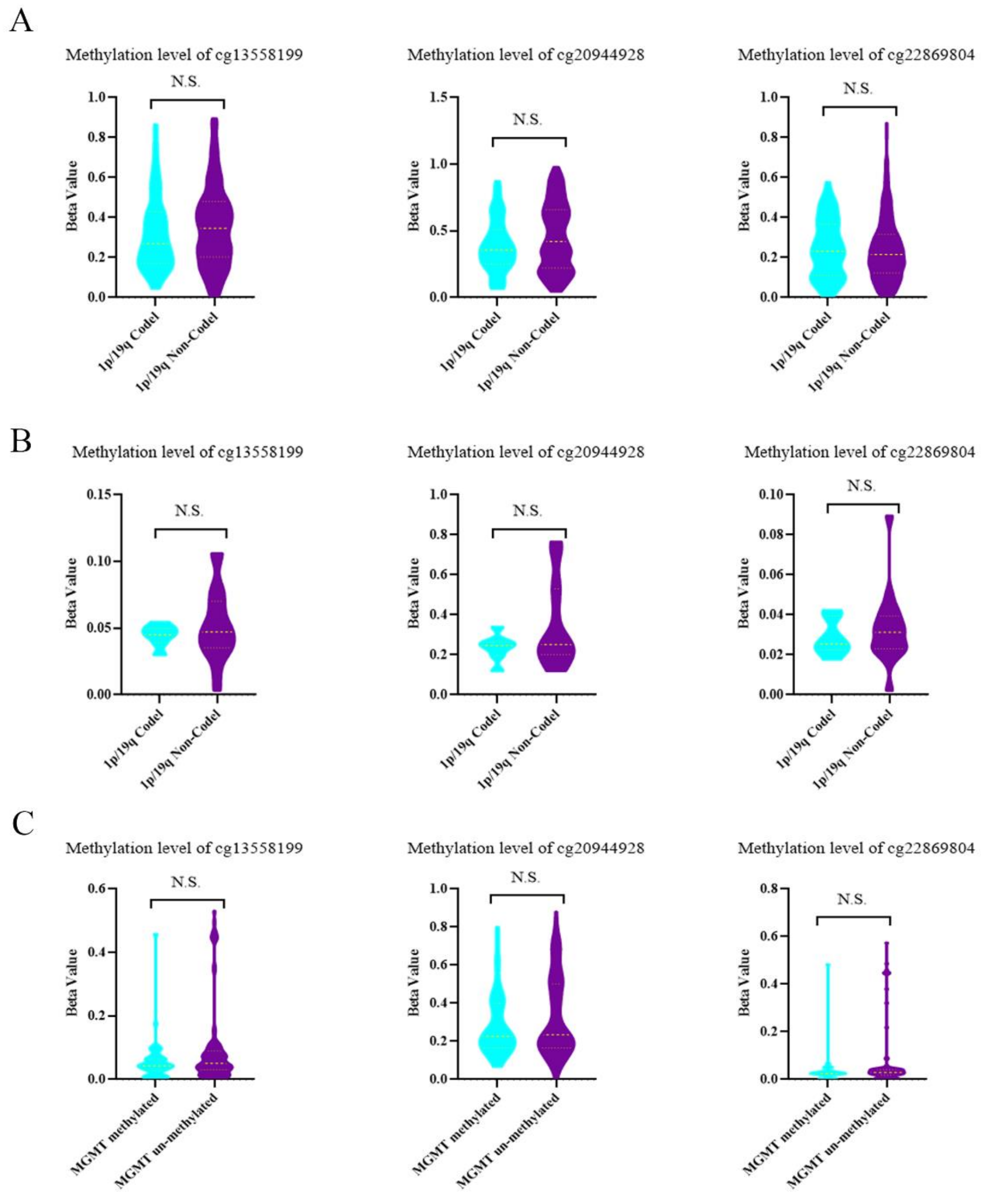

Supplementary figure 2. HIC2 is highly methylated in glioma cells

A, B: The gene location and primer sites for BGS sequence of HIC2 were visualized using the UCSC database and Methprimer database. **C:** Reduction and silencing of HIC2 in glioma cell lines. M: methylated; U: unmethylated. **D:** Representative BGS results of glioma cell lines. Filled circle representing methylated while open circles representing unmethylated CpG sites.

Supplementary figure 2

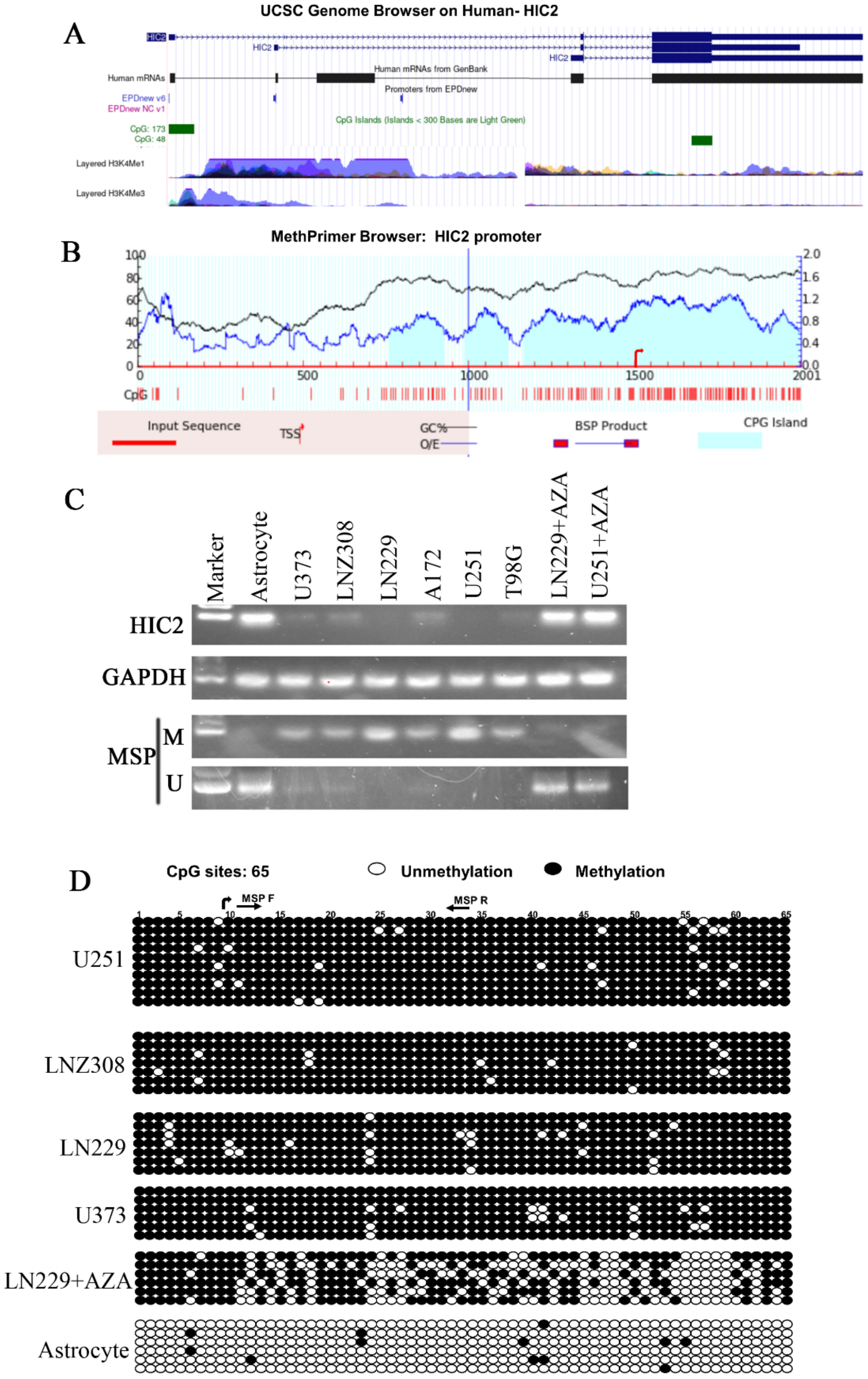

Supplementary figure 3: HIC2 expression is not different between MGMT promoter methylation group and MGMT promoter un-methylation group.

Supplementary figure 3

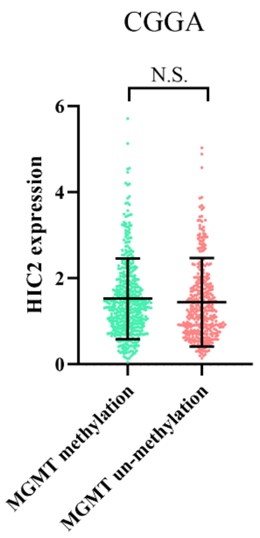

Supplementary figure 4. Cox regression analysis of HIC2 in the prognosis prediction of glioma patients

A: Univariate and multivariate Cox regression analyses based on data from CGGA. Left: univariate Cox regression; right: multivariate Cox regression. HR > 1 indicates disadvantageous factors; HR <1 indicates protective factors. **B:** Univariate and multivariate Cox regression analyses based on data from TGGA. **C:** A model describing the relationship between different risk scores and follow-up time, events, and changes in gene expression.

Supplementary figure 4

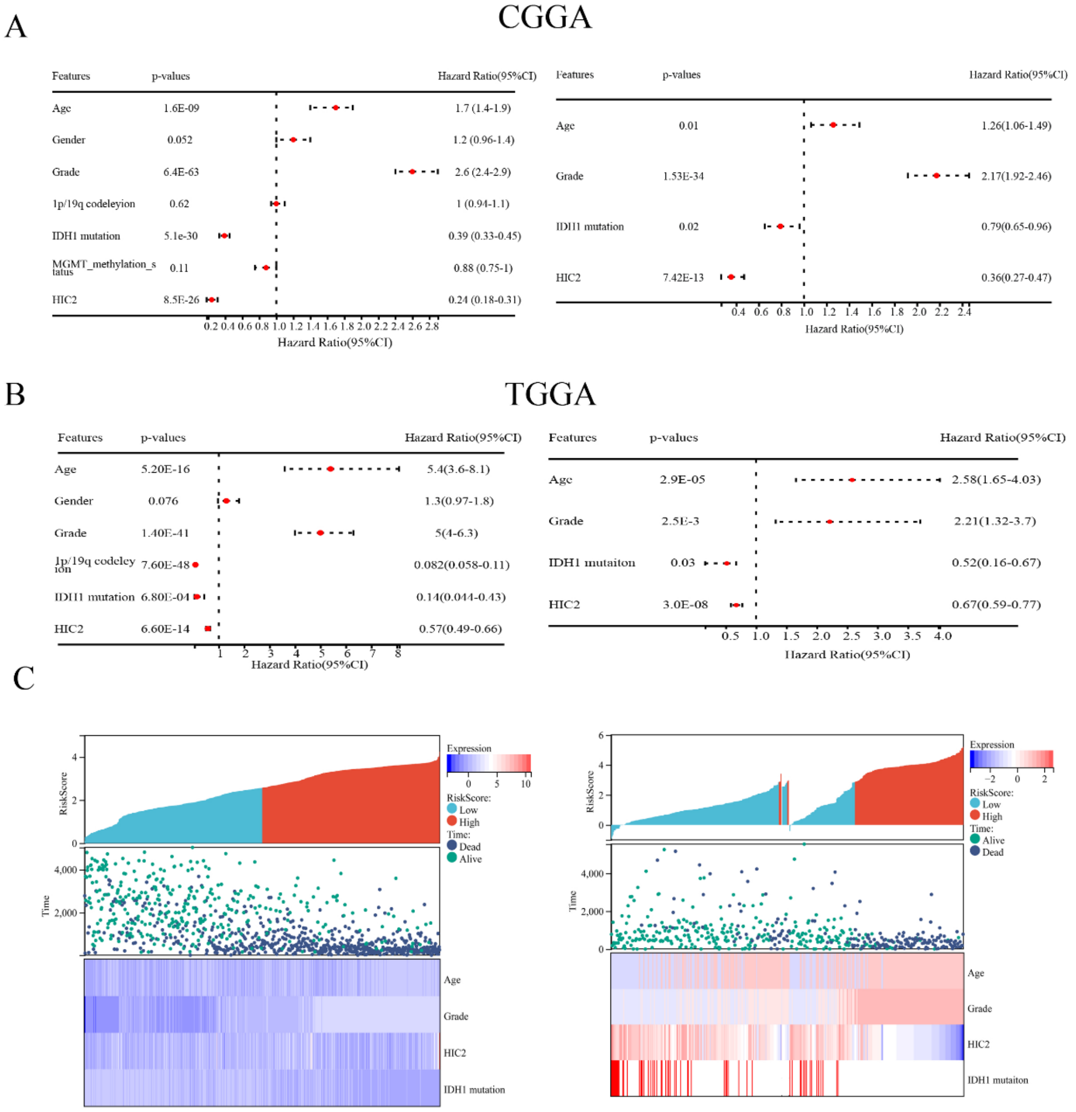

Supplementary figure 5. HIC2 is correlated with immune cell infiltration in glioma

A: The correlation between HIC2 expression and the infiltration level of six immune cell types based on data from LGG and GBM using the TIMER algorithm. **B:** The correlation between HIC2 expression and eight immune cell types based on data from CGGA and TCGA using the CIBERSORT algorithm. **C:** The correlation between DNA methylation levels at three CpG sites and eight immune cell types based on data from CGGA and TCGA using the CIBERSORT algorithm. **D:** High expression of HIC2 is negatively correlated with immune score. **E:** Heatmap of the correlation between the immune inhibitors and HIC2 expression in LGG and GBM (left); heatmap of the correlation between the immune stimulators and HIC2 expression in LGG and GBM (right). **F:** The correlation between HIC2 expression and immune checkpoints in the CGGA (up) and TCGA (down) datasets. **G-H:** The correlation between HIC2 expression and classical phenotype markers of macrophages (M0, M1 and M2) or neutrophils (N1, N2) in CGGA datasets (G) and TCGA datasets (H) (*: $p < 0.05$; **: $p < 0.01$; ***: $p < 0.001$ and ****: $p < 0.0001$; N.S.: not significant).

Supplementary figure 5

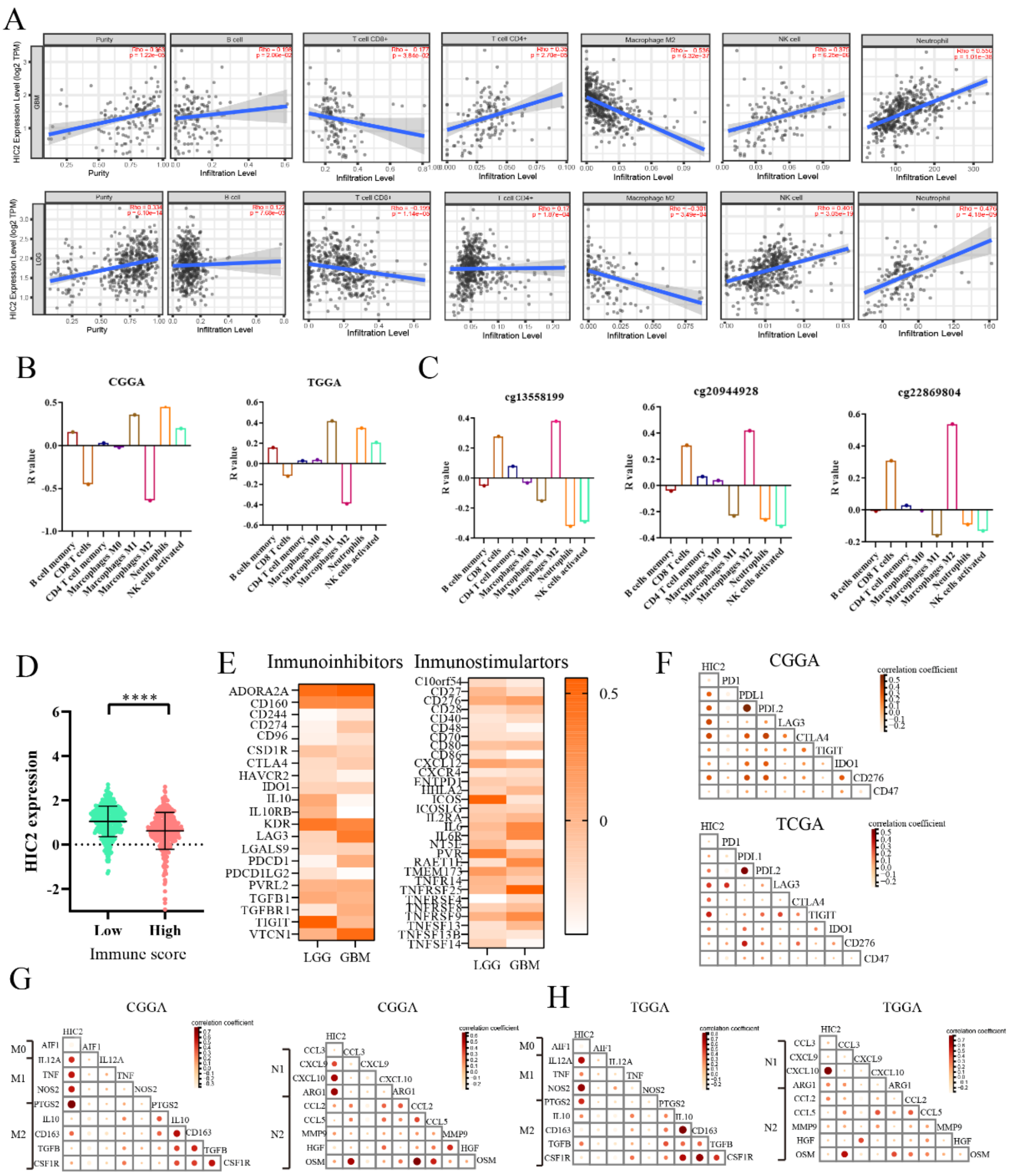

Supplement: Supplementary file 2 — Figure S1 Figure S2 Figure S3 Figure S4 Figure S5 [file CNS-29-1154-s001.pdf]
